# Draft genome of *Microsporidia* sp. MB—a malaria-blocking microsporidian symbiont of the *Anopheles arabiensis*

**DOI:** 10.1128/MRA.00903-23

**Published:** 2024-03-21

**Authors:** Lilian Mbaisi Ang'ang'o, Jacqueline Wahura Waweru, Edward Edmond Makhulu, Anne Wairimu, Fidel Gabriel Otieno, Thomas Onchuru, Özlem Tastan Bishop, Jeremy Keith Herren

**Affiliations:** 1 Research Unit in Bioinformatics (RUBi), Department of Biochemistry and Microbiology, Rhodes University, Makhanda, Eastern Cape, South Africa; 2 International Centre of Insect Physiology and Ecology (icipe), Nairobi, Kenya; University of Maryland School of Medicine, Baltimore, Maryland, USA

**Keywords:** microsporidia, anopheles, genomes, endosymbionts

## Abstract

We report the draft whole-genome assembly of *Microsporidia* sp. MB*,* a symbiotic malaria-transmission-blocking microsporidian isolated from *Anopheles arabiensis* in Kenya. The whole-genome sequence of *Microsporidia* sp. MB has a length of 5,908,979 bp, 2,335 contigs, and an average GC content of 31.12%.

## ANNOUNCEMENT

Microsporidia are microscopic, obligate intracellular eukaryotes that widely infect both vertebrates and invertebrates (1–7). *Microsporidia* sp. MB is a species of microsporidia that infects *Anopheles* mosquitoes and has been identified as a potential malaria transmission-blocking agent, as it can significantly reduce the vectorial capacity of *Anopheles* (8). Moreover, it exhibits positive effects on the fitness of its host, contributing to its spread in host populations (8, 9). Its unique characteristics and life cycle adaptations make it an intriguing subject for research in mosquito-borne disease control (10). We aimed to sequence and assemble the genome of this important symbiont isolated from *Anopheles arabiensis* mosquitoes in Kenya.

Gravid female mosquitoes were collected in Ahero (34.9190°W, −0.1661°N), Western Kenya and used to set up isofemale family lines. Genomic DNA was extracted from dissected ovaries of *Microsporidia* sp. MB-infected F1 progenies using the protein precipitation extraction protocol, as previously described, and screened for the symbiont using MB18S primers quantitative PCR assays (8). Highly infected samples were selected for sequencing after assessing DNA quality using Qubit Fluorometric Quantitation (ThermoFisher Scientific, Waltham, USA). Paired short-insert libraries were prepared using KAPA HiFi HotStart Library Amp Kit and sequenced with DNBSeq technology (2 × 150 bp reads) at BGI Genomics (https://www.bgi.com/global), generating a total of 326,181,200 raw paired-end reads. SOAPnuke v2.1.8 was employed to filter the reads using filtering parameters: “*-n 0.001 L 10 -q 0.5 --adaMR 0.25 --polyX 50 –minReadLen 100*” (11). FastQC v0.11.9 (https://www.bioinformatics.babraham.ac.uk/projects/fastqc/) (12) and MultiQC v1.12 (13) were used for quality assessment. The host reads were removed by mapping to the reference genomes of *A. arabiensis* (GenBank: GCA_000349185.1) and *A. gambiae s.s*. (GenBank: GCA_000005575.1) from the NCBI RefSeq database (release 219) (http://www.ncbi.nlm.nih.gov/RefSeq/) (14, 15) using the Burrows-Wheeler Aligner (BWA) v0.7.17 (https://github.com/lh3/bwa) (16). Samtools v1.3.1 (https://github.com/samtools/) (17) was used to filter out host-mapped reads. Kraken2 v2.0.8 (18) was applied to remove bacterial contaminants using the minikraken_8 GB_20200312 database. The clean reads were *de novo* assembled using Unicycler v0.4.9 (19), and a megablast search was conducted against microsporidia proteins to remove non-target contigs. The raw reads were reassembled to the cleaned assembly using BWA-MEM v0.7.17 (16) generating a consensus assembly. A remote BLAST against the NCBI nt database was used to identify contigs with high similarity to microsporidia. Gene prediction was performed using GeneMarkS v4.3 (20) (intronless eukaryotic mode), and RepeatModeler v2.0.4 (http://www.repeatmasker.org) (21) used to identify repeats in the assembly (RepBaseRepeatMaskerEdition-20181026). Genome completeness was assessed using Benchmarking Universal Single-Copy Orthologs (BUSCO) v5.4.3 (22) against the microsporidia_odb10 database (*n* = 600) (23) indicating 81% completeness. Quality assessment and genome statistics were determined using QUAST v5.2.0 (24), revealing a total genome size of 5.90898 Mb spanning 2,335 contigs, with an N_50_ of 5,000 bp (Table 1).

**TABLE 1:** *Microsporidia* sp. MB genome assembly statistics

Metric	
Assembly size (bp)	5,908,979
Number of contigs	2,335
N_50_ (bp)	5,000
GC content (%)	31.12
The proportion of repeats (%)	0.57
Number of predicted genes	2,247
Gene density (genes/kb)	0.363
Mean CDS length (bp)	1,108
BUSCO (*n* = 600)	
No. (%) of complete genes	486 (81.0)
No. (%) of complete and single-copy genes	485 (80.8)
Number (%) of complete and duplicated genes	1 (0.2)
Number (%) of fragmented genes	12 (2.0)
Number (%) of missing genes	102 (17.0)
GenBank accession number	JAVKTW000000000
SRA accession number	SRR25938329

**Fig 1:**
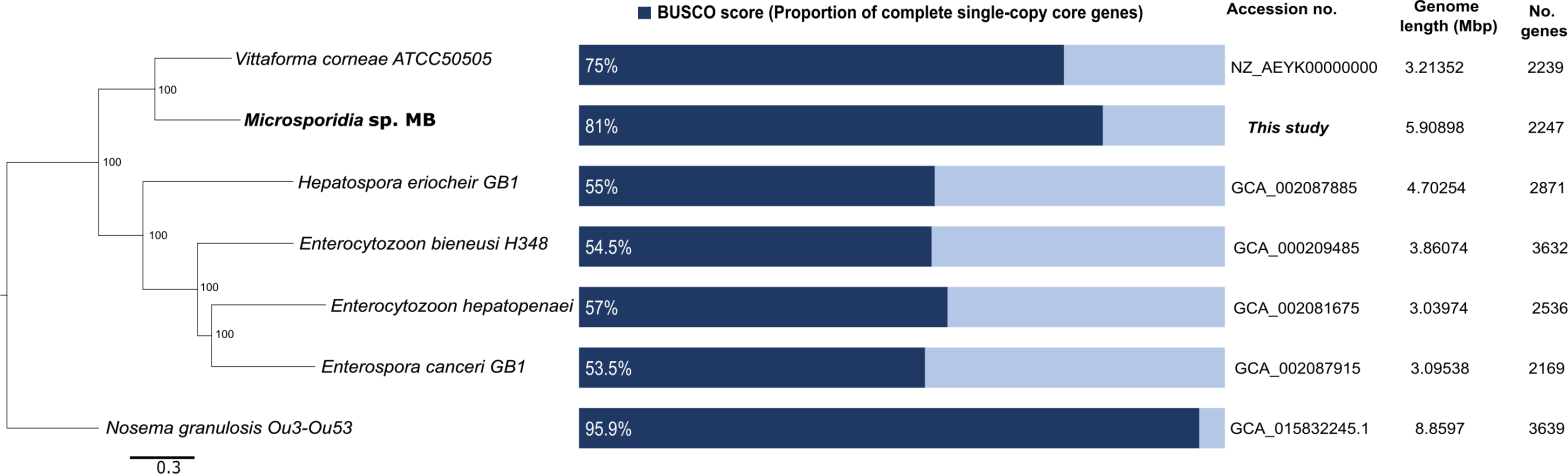
Phylogenomic analysis of *Microsporidia* sp. MB alongside genome assembly statistics comparison among the Enterocytozoonidae group of which five genomes have been fully sequenced. *Nosema granulosis* from the Nosematidae group of Microsporidia was used as an outgroup. The tree was constructed using maximum likelihood with FastTree v2 (26) based on the 399 single-copy orthologous genes found in all species using OrthoFinder v2.5.4 (25). Protein sequences were aligned with MAFFT v7 (27) using default options. Identified genes and species trees were generated on OrthoFinder v2.5.4 (28) and visualized on Dendroscope v3.8.4 (29). The phylogenomic tree reveals the close relationship between *Microsporidia* sp. MB and *Vittaforma corneae*.

Phylogenomic analysis using OrthoFinder v2.5.4 (25) showed *Microsporidia* sp. MB is closely related to *Vittaforma corneae* within the Enterocytozoonidae group, consistent with previous taxonomic classification based on SSU rRNA (4, 8, 10), and contained the highest proportion of core BUSCO genes (Fig. 1). Default parameters were used for all software except where otherwise noted.

## Data Availability

This Whole Genome Shotgun project has been deposited at DDBJ/ENA/GenBank under the accession number JAVKTW000000000. The version described in this paper is the first version, JAVKTW010000000. The raw reads have been deposited at SRA under the accession SRR25938329.
